# Comprehensive Assessment of High-Temperature Performance, Economic and Sustainability of MSWI Bottom Ash-Based Alkali-Activated Slag Paste

**DOI:** 10.3390/ma19102102

**Published:** 2026-05-16

**Authors:** Jingmei Wang, Yonghui Gao, Yifan Ma, Binbin Zhang, Yaoxiang Zhang, Yao Wang, Tao Ji

**Affiliations:** 1 School of Mathematics and Statistics, Shangqiu Normal University, Shangqiu 476000, China; 2 College of Smart City Engineering, Shangqiu Normal University, Shangqiu 476000, China; 3 School of Software, Shangqiu Normal University, Shangqiu 476000, China; 4 CSCEC Strait Construction and Development Co., Ltd., Fuzhou 350015, China; 5 School of Civil Engineering, Fuzhou University, Fuzhou 350108, China

**Keywords:** alkali-activated slag, MSWI-BA, economic viability, high-temperature resistance, environmental sustainability, carbon emission

## Abstract

This study presents a comprehensive assessment of high-temperature performance, economic viability, and environmental sustainability of alkali-activated slag paste (AASB) incorporating municipal solid waste incineration bottom ash (MSWI-BA). The research systematically evaluates the effects of MSWI-BA content (0–12%), alkali content (2–6% Na_2_O equivalent), water glass modulus (Ms = 0.75–1.75), and activator type on key performance metrics, both resource recovery and carbon reduction goals. Results show that the optimized formulation (6% MSWI-BA, 4% Na_2_O, Ms = 1.5) achieves superior high-temperature resilience, retaining 76% of its initial compressive strength after 800 °C exposure—a stark contrast to OPC, which undergoes near-complete strength loss. Economic analysis reveals that while MSWI-BA offers an 88% reduction in raw precursor cost, the optimized AASB incurs a modest 3.7% total material cost premium over OPC, which is offset by its long-term sustainability benefits. Furthermore, a life-cycle assessment demonstrates that AASB has a 66.95% lower carbon footprint than OPC.

## 1. Introduction

The global construction industry is currently navigating a critical transition toward sustainability, driven by the urgent need to mitigate carbon emissions and address the mounting challenges of solid waste management [1]. Ordinary Portland cement (OPC), the cornerstone of modern construction, is responsible for approximately 8% of global CO_2_ emissions due to clinker production, while simultaneously generating massive industrial by-products that strain waste disposal systems [2]. Concurrently, the rapid expansion of municipal solid waste incineration (MSWI) as a waste treatment strategy has led to the accumulation of MSWI bottom ash (MSWI-BA)—a by-product characterized by high volume, potential heavy metal leaching risks, and limited recycling pathways, posing significant environmental and economic burdens on urban ecosystems. This dual pressure—reducing carbon footprints while valorizing problematic waste streams—has spurred intensive research into alternative binder systems, with alkali-activated materials (AAMs) emerging as a promising low-carbon solution that leverages industrial by-products such as blast furnace slag (BFS) [3,4].

Alkali-activated slag systems have garnered attention for their ability to form stable calcium-aluminosilicate-hydrate (C-A-S-H) gels under alkaline activation, exhibiting mechanical properties comparable to OPC while eliminating clinker-related emissions [5]. This interest aligns with broader research on AAMs, which has been systematically reviewed in terms of reaction chemistry, binder microstructure, and long-term durability [6,7]. Recent efforts have extended beyond traditional BFS utilization to incorporate secondary waste materials, with MSWI-BA emerging as a particularly attractive candidate [8]. The utilization of MSWI-BA in cementitious systems has been extensively explored, with previous studies investigating its potential as both an aggregate and binder replacement in concrete and mortar applications [9]. MSWI-BA is rich in CaO and aluminosilicate components, confirming its reactivity in alkali-activated systems through X-ray diffraction (XRD) evidence of portlandite peaks and developing C-A-S-H phases [10]. Moreover, its economic advantage is compelling: with a unit cost of 5 USD/ton, MSWI-BA offers an 88% cost reduction compared to BFS (42.86 USD/ton), presenting opportunities to enhance the affordability of sustainable binders. Existing studies have explored MSWI-BA incorporation in alkali-activated systems, focusing primarily on basic workability and mechanical properties, yet critical knowledge gaps remain in holistic performance assessment (performance before and after high temperatures, sustainability evaluation).

While the individual merits of AAMs and MSWI-BA utilization have been extensively documented, studies combining these two strategies remain limited. Gao et al. [11] characterized the reaction process, gel composition, and microstructure of alkali-activated slag incorporating MSWI-BA and granite powder, demonstrating significant carbon emission reduction potential. Zhang et al. [12] demonstrated that MSWI-BA can enhance the high-temperature resistance of alkali-activated slag paste, while Huang et al. [13] investigated the polymerization mechanism and strength development of MSWI bottom ash alkali-activated mortars with activated silica. However, these studies primarily focused on basic workability and mechanical properties at ambient conditions, with systematic evaluations of high-temperature performance, economic viability, and environmental sustainability—particularly under multi-variable optimization—remaining notably absent.

Three key research domains require more in-depth investigation. First, high-temperature performance—a critical attribute for structural and fire-resistant applications—remains underexplored for MSWI-BA-incorporated alkali-activated slag pastes. While the high-temperature behavior of alkali-activated fly ash and slag systems has been systematically characterized [14,15], demonstrating superior thermal stability compared to OPC due to the absence of Ca(OH)_2_ decomposition, the specific effects of MSWI-BA incorporation on thermal degradation pathways, mass loss kinetics, and residual mechanical strength remain poorly understood. While AAMs generally outperform OPC at elevated temperatures by avoiding catastrophic strength loss from portlandite decomposition (400–450 °C) [16,17], the effects of MSWI-BA on high-temperature mass loss, thermal shrinkage, and residual mechanical strength under varied thermal regimes (25–1000 °C) lack systematic assessments. Previous life-cycle assessments of alkali-activated materials have consistently demonstrated significant carbon reduction potential compared to OPC [18,19], with some studies integrating economic indicators to evaluate multi-objective sustainability [20]. However, these assessments have predominantly focused on conventional precursors (fly ash, slag) without considering the specific valorization impacts of MSWI-BA, including avoided landfilling emissions and heavy metal immobilization benefits. Second, economic evaluations of such systems have typically focused on material costs alone, neglecting comprehensive analyses of life-cycle viability, including waste disposal savings, activator production economics, and scalability potential [21]. Third, environmental sustainability assessments, although acknowledging carbon reduction benefits, often lack quantitative integration of MSWI-BA valorization impacts (e.g., reduced landfilling emissions, heavy metal immobilization) alongside carbon footprint analyses. In recent years, a series of LCA studies have verified that alkali-activated materials present obvious advantages in carbon emission reduction and energy saving compared with OPC, providing important support for the sustainable development of low-carbon binders. Furthermore, the synergistic effects of multi-variable parameters—such as MSWI-BA content, alkali content, water glass (WG) modulus, and activator type—on the interplay between high-temperature performance, economic viability, and environmental benefits remain poorly understood.

This study presents a comprehensive assessment of MSWI-BA-incorporated alkali-activated slag paste (AASB), focusing on three interconnected dimensions: high-temperature performance, economic viability, and environmental sustainability. By systematically evaluating the effects of MSWI-BA content (0–12%), alkali content (2–6% Na_2_O equivalent), WG modulus (Ms = 0.75–1.75), and activator type (sodium vs. potassium-based) on thermal stability (mass loss, shrinkage, and residual strength), material cost structures, and life-cycle environmental impacts (carbon footprint, waste valorization), this research aims to identify optimized formulations that balance technical performance, affordability, and sustainability. The findings of this study are expected to provide critical insights for advancing MSWI-BA as a high-value resource in low-carbon construction, bridging the gap between waste management, emissions reduction, and high-performance material development.

## 2. Materials and Methods

### 2.1. Raw Materials

Blast furnace slag (BFS) and municipal solid waste incineration bottom ash (MSWI-BA) were used as main precursor materials. MSWI-BA (5 USD/ton) reduces raw material costs by 88% compared with BFS (42.86 USD/ton) [12] and can be incorporated up to 12% without obvious performance degradation.

**Figure 1:**
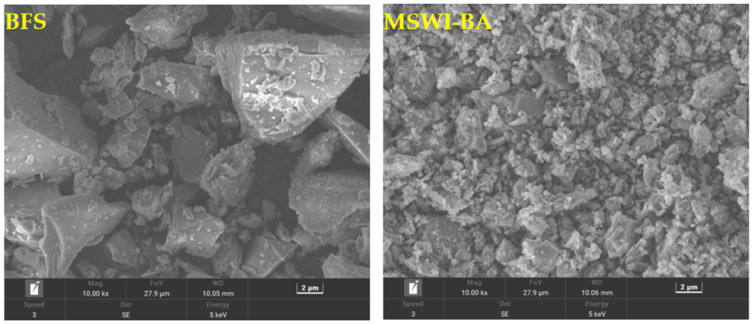
SEM patterns of precursor [12].

**Figure 2:**
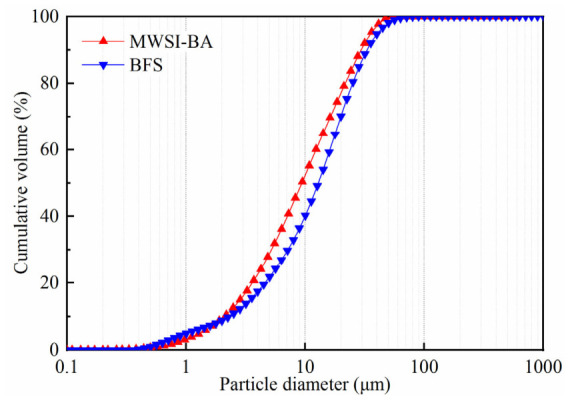
Particle size distributions of precursor [12].

**Figure 3:**
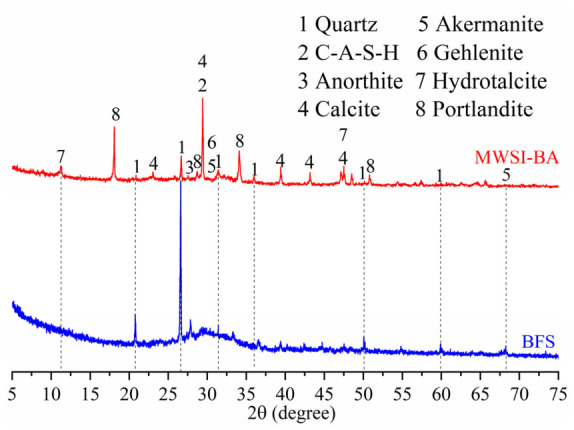
XRD patterns of precursor [12].

The chemical composition of raw materials was quantitatively analyzed using X-ray fluorescence spectroscopy (Table 1), while physical property measurements (Table 2) revealed that MSWI-BA exhibits significantly higher water absorption and specific surface area compared to BFS, primarily due to its inherent porous structure [12]. SEM images (Figure 1) clearly demonstrate the contrasting morphologies, with MSWI-BA displaying irregular, highly porous particles versus the dense, smooth surfaces of BFS particles. Particle size distribution analysis (Figure 2) further highlights these differences in material characteristics. XRD patterns (Figure 3) identified distinct phase compositions, with MSWI-BA showing characteristic portlandite peaks (resulting from CaO hydration) alongside developing C-A-S-H and hydrotalcite phases, confirming active alkali activation of its aluminosilicate components [22]. This activation mechanism not only facilitates large-scale utilization of MSWI-BA but also effectively immobilizes heavy metals and chlorides through chemical incorporation in the aluminosilicate matrix [23], demonstrating its dual environmental and technical benefits in sustainable binder applications.

The alkaline activator solutions were formulated by adjusting commercial sodium silicate (26.5 wt% SiO_2_, initial modulus Ms = 3.3) with analytical-grade sodium hydroxide (NaOH, ≥99% purity) to obtain desired WG moduli (Ms = 0.75–1.75). The modification process involved: (i) precise dissolution of NaOH pellets in deionized water to form concentrated alkaline solutions, (ii) controlled mixing with sodium silicate under continuous mechanical stirring (500 rpm, 30 min), and (iii) sealed storage in containers for a minimum 24 h equilibration period to ensure complete silicate species polymerization and solution homogeneity. This standardized preparation protocol guaranteed consistent activator chemistry across all experimental groups while preventing carbonation through rigorous container sealing.

### 2.2. Mix Proportions

The experimental variables and levels were determined based on preliminary tests and literature [12]: (1) MSWI-BA was set at 0–12% because beyond 12% significant workability loss and strength reduction occur; (2) Na_2_O equivalent was selected as 2–6% to cover insufficient and excessive activation ranges; (3) water glass modulus Ms = 0.75–1.75 was chosen to reflect low, medium and high silicate conditions; (4) four activator types (sodium- vs. potassium-based) were compared to reveal cation effects. One variable was changed while others were fixed at the reference level (6% MSWI-BA, 4% Na_2_O, Ms = 1.5, NN activator) to ensure independent variable analysis.

The AASB paste formulations are summarized in Table 3 and Table 4. The number after the label “NN-” represents the MSWI-BA content, which is the percentage of precursors (BFS + MSWI-BA). The number after the label “NN” represents the Na_2_O content. The Na_2_O equivalent is the percentage content of the mass of precursors. The number after “Ms” on the label represents the modulus of Na water glass after being modulated with NaOH. The water-binder ratio is 0.35, which is the ratio of total water (added water and water in WG) to cementitious materials (precursors and solid components in sodium silicate). OPC with the same water-binder ratio is set as the control group. The OPC control group used ordinary Portland cement (P·O 42.5) with the same water-binder ratio (0.35) and no other admixtures, and was cured under the same conditions as AASB samples.

### 2.3. Sample Preparation

#### 2.3.1. Mixing Procedure

Raw materials were mixed in a standard cement mortar mixer (JJ-5 type, batch volume 1.5 L). Dry precursors were pre-blended for 1 min to achieve uniformity. Simultaneously, the WG activator was combined with the full amount of designated mixing water to create a consistent alkaline solution. The mixing process employed a sequential addition method: initially, half of the prepared alkaline solution was poured into the dry material mixture and blended for one minute, after which the residual 50% solution was slowly added. The resulting composite was then subjected to a final 60 s mechanical mixing cycle to guarantee full homogenization and appropriate rheological properties of the freshly prepared alkali-activated slag binder.

#### 2.3.2. Casting and Curing

The freshly prepared AASB was molded using a sequential filling approach, where each layer underwent 60 s of vibration densification on a calibrated vibration table. Two specimen configurations were produced: prismatic samples (40 × 40 × 160 mm) for mechanical strength testing and elongated bars (25 × 25 × 285 mm) for thermal deformation analysis. Following molding, all test pieces were immediately wrapped in airtight plastic sheeting and stored at 25 ± 2 °C. After 24 h of initial curing, specimens were demolded and cured in a chamber (20 ± 2 °C, 95% RH) for 28 days before thermal treatment and performance testing.

### 2.4. Test Methods

#### 2.4.1. Workability

The rheological properties of freshly prepared mixtures were assessed according to standardized protocols. Flow characteristics were measured using a precision-engineered flow cone apparatus conforming to GB/T 2419-2005 specifications [24], featuring exacting dimensional tolerances (60 ± 0.5 mm height, 70 ± 0.5 mm upper aperture, 100 ± 0.5 mm base diameter). The testing protocol involved filling the mold, carefully extracting it, and executing 25 controlled table drops within a strict 25 ± 1 s timeframe to quantify the mortar spread diameter. In parallel, hydration kinetics were investigated through Vicat apparatus testing per GB/T 1346-2011 guidelines [25]. The reduction in needle penetration depth over time reflects the setting and rheological behavior of the paste.

#### 2.4.2. Heat Treatment

**Figure 4:**
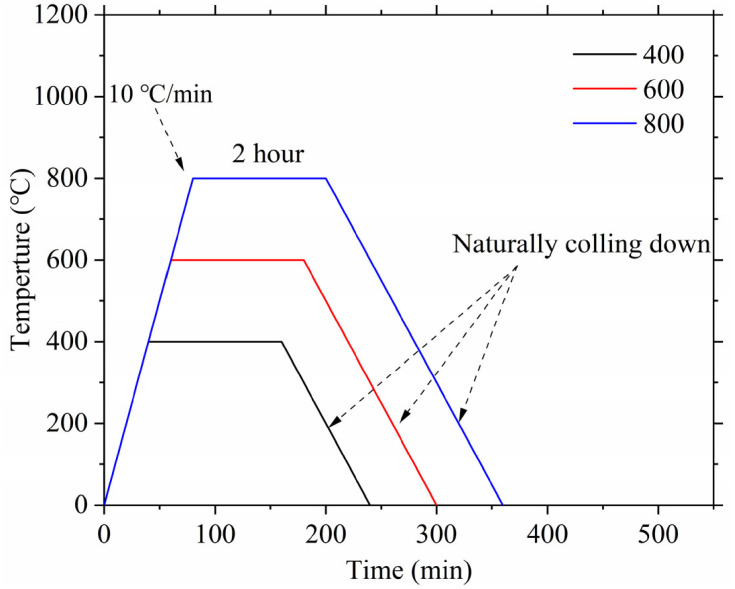
Heating regimes for AASB [12].

The high-temperature resistance of cured specimens was systematically examined after 28 days of standard hydration using an electrically controlled furnace with standardized thermal protocols [12]. The experimental thermal regime incorporated a precisely controlled heating phase from room temperature to designated evaluation temperatures (400 °C, 600 °C, 800 °C) at a fixed heating rate of 10 °C per minute. Each target temperature was maintained for a 120-min isothermal period to guarantee uniform heat distribution and thermal stabilization within the samples. Following the heating cycle, specimens were cooled naturally within the furnace environment to prevent thermal shock, with the entire thermal profile (detailed in Figure 4) benchmarked against the RILEM TC 129-MHT recommendations for concrete at high temperatures, carefully designed to facilitate accurate assessment of temperature-induced material transformations while ensuring experimental reproducibility.

#### 2.4.3. Mass Loss and Thermal Shrinkage

Thermal stability was evaluated using 40 × 40 × 40 mm cubic specimens for mass loss and 25 × 25 × 285 mm bar specimens for linear shrinkage, measured by a calibrated analytical balance (±0.01 g) and digital comparator (±0.001 mm), respectively. Following standardized protocols, each sample was weighed before (M0) and after (M1) thermal treatment, with mass loss percentage (ΔM) calculated according to Equation (1). To ensure data reliability, three parallel samples were prepared for each test, and all measurements were performed in triplicate under controlled laboratory conditions. The results are presented as average values, and test variability was maintained within ±2% through rigorous calibration procedures. The results represent arithmetic means of three independent determinations, demonstrating excellent reproducibility across multiple test series.

Linear dimensional changes were quantified using a high-precision digital comparator (±0.001 mm accuracy) per ASTM C490 [26]. Specimen lengths were recorded before (L0) and after (L1) thermal exposure, with thermal shrinkage (ΔL) calculated according to Equation (2). The testing protocol incorporated multiple quality control measures, including triplicate measurements, reference-grade calibration standards, and strict environmental controls. Experimental variability was constrained to ±0.5% through standardized operator training and randomized testing sequences, ensuring statistically significant results for all evaluated formulations. This systematic approach enabled accurate characterization of temperature-induced dimensional changes while maintaining exceptional measurement reproducibility.

ΔM = [(M0 − M1)/M0] × 100%,(1)

ΔL = [(L0 − L1)/L0] × 100%(2)

#### 2.4.4. Residual Strength

The mechanical behavior of cured specimens was characterized through comprehensive bending and compression testing following standardized protocols. Flexural strength assessment was conducted on prismatic samples (40 × 40 × 160 mm) using a computer-controlled electrohydraulic testing machine (DYE-2000 series) in accordance with GB/T 17671-1999 [27]. The three-point bending tests were performed at a constant stress rate of 0.09 ± 0.01 MPa/s, with each formulation group tested in triplicate to ensure statistical reliability. Following flexural failure, the resulting fragments (40 × 40 mm) were immediately subjected to uniaxial compression testing at a controlled loading rate of 1.3 ± 0.1 MPa/s, generating six independent measurements per mixture design. All mechanical testing was conducted under strictly controlled environmental conditions, with specimen alignment verified using precision leveling instruments prior to each test. All residual strength results were normalized by the 28-day compressive/flexural strength of unheated control specimens from the same mix to quantify high-temperature degradation.

## 3. Results

### 3.1. Workability

**Figure 5:**
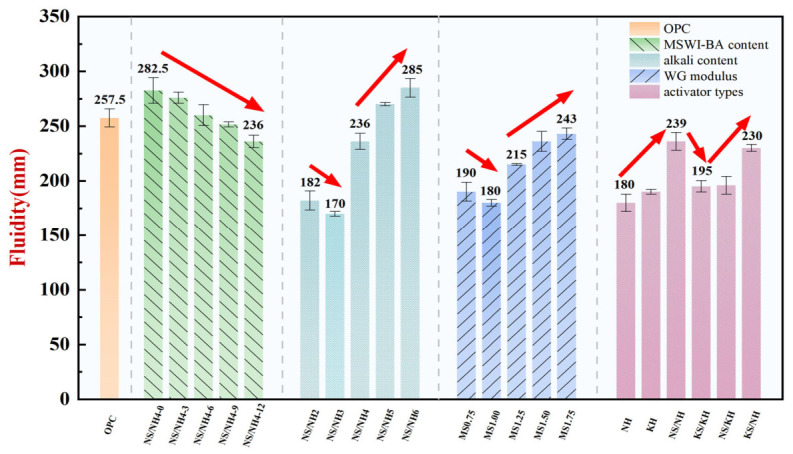
Fluidity of OPC and AASB under different variables (Error bars: SD of 3 parallel tests).

Figure 5 presents a systematic comparison of workability (quantified by flow diameter) between AASB binders and OPC, evaluating four critical variables: MSWI-BA content, alkali content, WG modulus, and activator type. The incorporation of MSWI-BA from 0% to 12% resulted in a proportional decrease in flow diameter from 283 mm to 236 mm (16.7% reduction). This observed linear trend is mechanistically attributed to the inherently elevated specific surface area and angular particle morphology characteristic of MSWI-BA [28]. In contrast, alkali content (2–6% Na_2_O eq.) and WG modulus (0.75–1.75) exhibited non-linear U-shaped responses. Minimum fluidity occurred at 3% alkali content and 1.0 WG modulus, attributed to accelerated dissolution of precursors (Ca^2+^, SiO_4_^4−^, AlO_4_^5−^) at intermediate concentrations, promoting rapid supersaturation and formation of a dense, high-viscosity C-(A)-S-H gel network [29]. Activator type further modulated workability: NH yielded the poorest fluidity (180 mm), while NN achieved superior fluidity (236 mm) due to soluble silicates enhancing colloidal stability via electrostatic repulsion and reduced inter-particle attraction [30].

The workability of AASB is governed by a complex interplay of competing physicochemical mechanisms. The inherent physical water demand of MSWI-BA and slag particles—attributed to their high specific surface area and irregular morphology—counterbalances the fluidizing effects of chemical activators. At low alkali content/WG modulus values, limited dissolution restricts gel formation, preserving workability; at intermediate values, the reaction rate is relatively fast, and the structural development of the paste is obvious; at high values, excess OH^−^ or SiO_4_^4−^ alters gel nanostructure (e.g., increased porosity or reduced cross-linking density) or induces deflocculation, partially restoring flow [31]. The enhanced performance of the NN activator can be attributed to the dual functionality of soluble silicates, which serve as both reactive components and effective dispersants. Specifically, these silicates adsorb onto precursor particle surfaces, forming electrostatically stabilized double layers that effectively prevent particle agglomeration through interparticle repulsion forces [32]. This mechanistic interpretation is further corroborated by recent molecular dynamics simulations demonstrating the capability of silicate species to significantly reduce interfacial energy between adjacent particles, thereby promoting colloidal stability and enhancing reactivity [33].

Under identical water-to-binder ratio conditions, the experimental results demonstrated that the NN-6 mix with 6% MSWI-BA content achieved superior workability (260 mm flow diameter) compared to conventional OPC (257 mm), while other formulations (NN-4, Ms1.5, and NN) maintained comparable performance (236 mm). These findings contrast with previous reports [34], which indicated significant workability reduction when incorporating MSWI-BA, primarily attributed to the material’s irregular particle morphology, higher water absorption capacity, and increased specific surface area [35]. The enhanced performance observed in this study stems from the optimized NN activator system, featuring (i) a carefully balanced alkali content (4% Na_2_O eq.) that prevents both insufficient activation and premature setting and (ii) an intermediate modulus (Ms = 1.5) that ensures adequate reactive silica availability while regulating dissolution kinetics. This demonstrates that appropriate activator design can effectively overcome the MSWI-BA incorporation threshold while maintaining or even improving upon OPC-equivalent workability characteristics.

**Figure 6:**
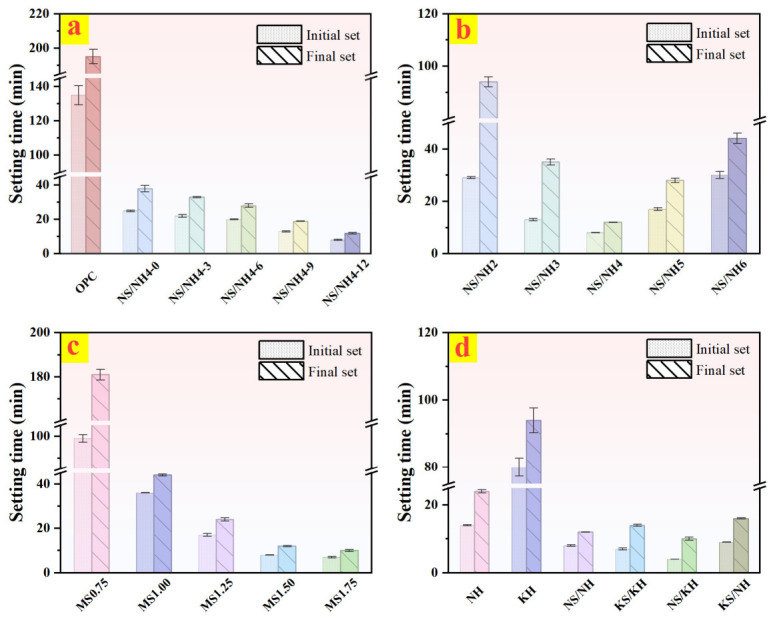
Setting time of OPC and AASB under different variables. (**a**) MSWI-BA content; (**b**) Alkali content; (**c**) WG moduli; (**d**) Activator types.

Figure 6 illustrates the comparative initial and final setting times of AASB and OPC under varying conditions. When other parameters were held constant, increasing the MSWI-BA content from 0% to 12% reduced both initial and final setting times by 8 min and 12 min, respectively, corresponding to reductions of 68.00% and 68.40% relative to the maxima (25 min and 38 min). This acceleration is attributed to the enhanced reactivity of MSWI-BA particles at higher dosages, which promotes rapid nucleation of C-A-S-H and hydrotalcite-like phases, thereby shortening the induction period [36]. In contrast, OPC typically exhibits setting times exceeding 45 min for initial setting and >375 min for final setting under standard conditions, highlighting the superior early-age reactivity of AASB systems.

The relationship between alkali content (2–4% Na_2_O equivalent) and setting behavior exhibited a distinct U-shaped trend, with minimum setting times observed at intermediate alkali concentrations (8 min initial, 12 min final for NN4 formulation). This non-linear response reflects two competing mechanisms: (i) insufficient MSWI-BA dissolution at low alkali concentrations (<3% Na_2_O eq.) due to limited availability of hydroxyl ions and (ii) ionic strength effects at high alkali concentrations (>4% Na_2_O eq.) that disrupt the polymerization of silicate species.

A more pronounced effect was observed with variation in WG modulus (Ms = 0.75–1.75), where increasing silicate content reduced setting times by 92.93% (initial) and 94.48% (final). This acceleration correlates with the enhanced availability of reactive silicate oligomers (particularly Q^2^ and Q^3^ species) that facilitate rapid C-A-S-H gel nucleation [37]. The fastest setting kinetics were achieved in NK-activated systems, with setting times ranging from 4 to 80 min (initial) to 10–94 min (final). This superior performance stems from the dual activation mechanism combining high pH dissolution (from NaOH) with network-forming silicate species (from Na_2_SiO_3_) [38].

### 3.2. Mass Loss

**Figure 7:**
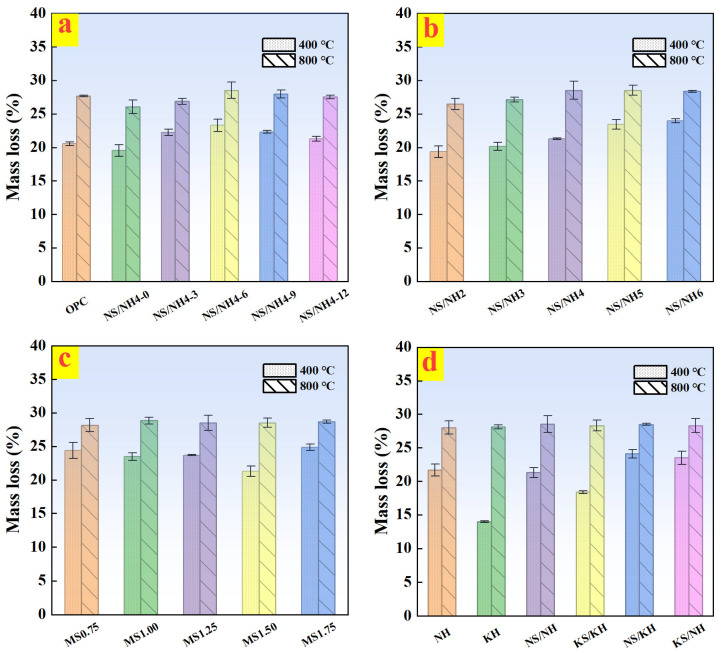
Mass loss (400 °C and 800 °C) of OPC and AASB under different variables. (**a**) MSWI-BA content; (**b**) Alkali content; (**c**) WG moduli; (**d**) Activator types.

Figure 7 presents a comparison of the mass loss between AASB binders and OPC at elevated temperatures. In civil engineering fire-resistant design, 400 °C and 800 °C are selected as typical critical temperatures. The temperature of 400 °C corresponds to the decomposition temperature of Ca(OH)_2_ in OPC, while 800 °C represents the severe high-temperature condition of structural components in fire. Thus, performance comparisons at 400 °C and 800 °C are mainly analyzed. The results demonstrate that AASB systems exhibit complex, composition-dependent behavior at 400 °C, with mass loss patterns varying significantly across different formulations. Increasing MSWI-BA content from 0% to 12% produced a non-monotonic response, peaking at 6% MSWI-BA incorporation (NN-6 group), which reflects the competing effects of enhanced chemically bound water content in C-A-S-H and hydrotalcite phases at moderate MSWI-BA contents versus improved cross-linking density at higher dosages [12]. Alkali content showed a direct positive correlation with mass loss at 400 °C, attributable to the formation of thermally unstable C-A-S-H gels at elevated alkali concentrations [39]. The WG modulus exhibited a characteristic U-shaped relationship, where intermediate SiO_2_/Na_2_O ratios promoted optimal silicate polymerization and matrix densification [38]. Notably, activator chemistry played a crucial role, with potassium-based (KH) systems demonstrating superior thermal stability (13.97% mass loss) compared to sodium-based formulations (up to 24.09% loss), highlighting the stabilizing effect of K^+^ ions on aluminosilicate networks [40].

At the more severe 800 °C exposure, all AASB formulations converged to approximately 28% mass loss, indicating complete decomposition of hydration products and carbonate phases. This consistent behavior across different compositions suggests the dominance of thermodynamic stability over mix design variables at extreme temperatures, with the observed mass loss corresponding to established dehydroxylation and amorphous-to-crystalline transition processes in geopolymer systems [41]. When compared with conventional OPC, which typically shows 15–20% mass loss at 400 °C (primarily from portlandite decomposition) and 20–25% at 800 °C (due to decarbonation reactions), AASB systems demonstrated comparable or superior thermal resistance. The exceptional performance of potassium-activated systems at 400 °C, coupled with the general convergence of all formulations’ behavior at 800 °C, provides important insights into the thermal stability limits of AAMs [42]. These findings have significant implications for the development of fire-resistant construction materials, particularly in applications where thermal performance is critical.

### 3.3. Thermal Shrinkage

**Figure 8:**
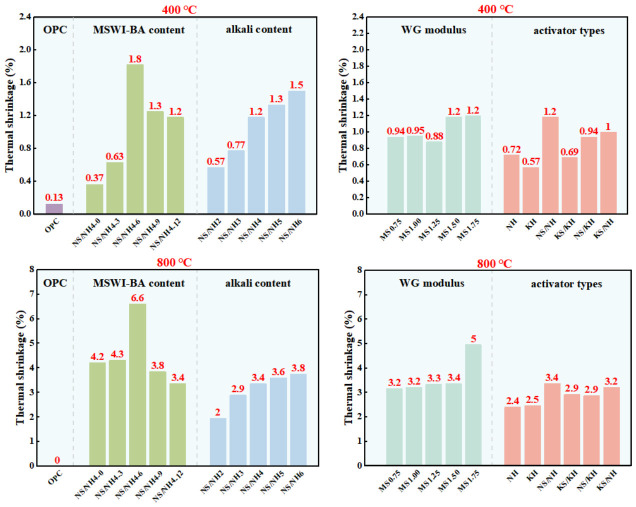
Thermal shrinkage of OPC and AASB under different variables.

Figure 8 presents the thermal shrinkage of AASB and OPC after exposure to 400 °C and 800 °C, revealing a consistent trend with mass loss attributed to dehydration and decomposition of hydration products. Under fixed variables, increasing MSWI-BA content from 0% to 12% induced a non-monotonic response in thermal shrinkage at both temperatures, peaking in the NN-6 group. This arises from the dual role of MSWI-BA: moderate dosages enhance cross-linking in C-A-S-H gel, reducing shrinkage, while excessive MSWI-BA dilutes the binder matrix, amplifying thermal deformation. Elevating alkali content from 2% to 6% monotonically increased shrinkage at 400 °C and 800 °C, correlating with accelerated formation of metastable sodium aluminosilicate hydrate (N-A-S-H) gel, which undergoes significant syneresis and pore collapse during heating [43]. The WG modulus exhibited divergent effects: shrinkage at 400 °C followed a U-shaped curve (minimal at intermediate moduli due to optimized polymerization), while at 800 °C it rose continuously—likely due to excess silica promoting viscous sintering and densification. Activator type critically influenced performance, with NaOH-activated systems (NN) showing the highest shrinkage, whereas KOH-based systems (KH) demonstrated superior stability, consistent with potassium’s role in mitigating capillary tension [44].

Notably, AASB exhibited greater thermal shrinkage than OPC at both temperatures under identical water-to-binder ratios. This differential response originates from distinct dehydration mechanisms in their respective hydration products. The dominant C-A-S-H gel phase in AASB experiences progressive water loss between 25 and 250 °C, involving both interlayer water removal and structural hydroxyl decomposition, which collectively contribute to early-stage matrix contraction [45]. OPC systems, conversely, benefit from the presence of approximately 20% portlandite (Ca(OH)_2_), which undergoes endothermic decomposition at 400–450 °C, generating expansive calcium oxide that temporarily offsets shrinkage forces and delays densification [46]. Quantitative measurements demonstrate this performance gap, with OPC exhibiting 400 °C shrinkage values of 0.3–0.8% compared to 0.57–1.18% for AASB, consistent with established literature documenting OPC’s superior dimensional stability below 500 °C [47]. At 800 °C, both systems converge toward maximum shrinkage thresholds as complete decomposition of all hydrated phases (including C-(A)-S-H and portlandite) becomes the dominant mechanism, overriding initial compositional differences. These findings highlight an inherent performance trade-off in AASB systems, while their rapid gel formation kinetics promote excellent early-age strength development, the resulting amorphous microstructure provides less resistance to high-temperature dimensional changes compared to OPC’s crystalline phase-stabilized matrix.

### 3.4. Residual Strength

**Figure 9:**
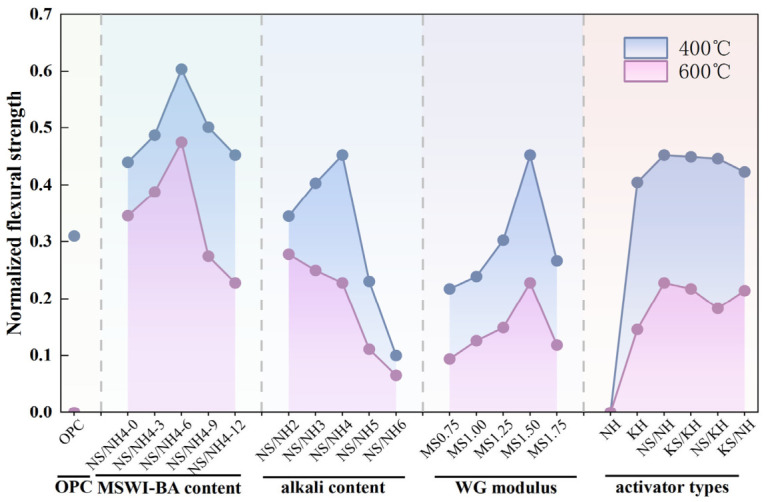
Normalized flexural strength of OPC and AASB under different variables.

Figure 9 compares the normalized flexural strength of AASB and OPC after exposure to 400 °C and 600 °C, excluding 800 °C due to complete strength loss. Under fixed variables, increasing MSWI-BA content from 0% to 12% induced a non-monotonic response; normalized flexural strength peaked in the NN-6 group at both temperatures. This optimum stems from MSWI-BA’s dual role—moderate dosages enhance pore connectivity and refine the matrix, improving thermal stability by mitigating crack propagation, while excessive MSWI-BA dilutes the binder phase, increasing porosity and reducing strength retention. Similarly, alkali content and WG modulus exhibited inverted U-shaped trends, with maxima in the NN4 and Ms1.50 groups, respectively. The alkali optimum (4%) balances accelerated geopolymerization (boosting early strength) against excessive Na^+^-induced microcracking during heating. The modulus optimum (1.50) aligns with maximized cross-linking of aluminosilicate networks, whereas higher moduli introduce unreacted silica, creating weak interfaces [48]. Activator type significantly influenced performance: NH-activated systems showed zero strength due to volatile decomposition, while NN systems achieved the highest residual strength, attributed to stable N-A-S-H formation.

Comparative analysis reveals that optimized AASB formulations (2–4% alkali content, modulus = 1.5) exhibit superior normalized flexural strength retention versus OPC at both 400 °C and 600 °C, fundamentally attributed to their distinct hydration product stability characteristics. While OPC suffers catastrophic strength loss due to expansive decomposition of its ~20% Ca(OH)_2_ content (400–450 °C), which creates microcracking and reduces strength to zero by 600 °C, AASB systems maintain structural integrity through gradual dehydration of their dominant C-A-S-H/N-A-S-H gel phases without disruptive phase transformations [49]. This critical difference stems from AASB’s avoidance of Ca(OH)_2_ formation, thereby eliminating a key weakness in OPC where its decomposition promotes crack coalescence. Notably, even non-optimized AASB specimens (excluding NH-activated systems) retained measurable strength at 600 °C, contrasting sharply with OPC’s complete failure, consistent with established reports of >50% strength retention in geopolymers versus near-total OPC degradation at similar temperatures. These findings underscore AASB’s significant potential for high-temperature applications where conventional cementitious materials are fundamentally limited by their calcium hydroxide content.

**Figure 10:**
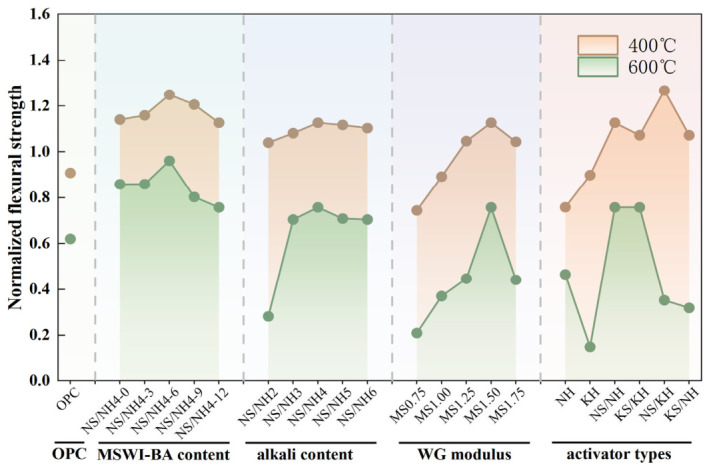
Normalized compressive strength of OPC and AASB under different variables.

Figure 10 demonstrates the normalized compressive strength evolution of AASB relative to OPC, revealing several key composition–performance relationships. The data show a non-monotonic relationship between MSWI-BA content and normalized compressive strength at both 400 °C and 800 °C, with optimal performance observed at 6% MSWI-BA incorporation (NN-6). This peak performance results from competing mechanisms: (i) enhanced matrix densification and pore refinement at moderate MSWI-BA contents versus (ii) increased porosity and binder phase dilution at higher dosages. Similar optimization trends emerged for alkali content and WG modulus, with maxima observed at 4% Na_2_O equivalent (NN4) and modulus 1.50 (MS1.50), respectively. The 4% alkali optimum represents an ideal balance between complete precursor activation and avoidance of excessive Na^+^-induced microcracking during thermal exposure, while the modulus optimum (1.50) provides optimal cross-linking density without generating weak interfaces from unreacted silica. Activator chemistry played a decisive role, with NH failing due to thermal decomposition, while NN combinations achieved exceptional residual strength (normalized values of 1.26 at 400 °C and 0.76 at 800 °C) through synergistic stabilization of N-A-S-H gels. These findings highlight the importance of precise composition control in AASB systems, with optimized formulations outperforming OPC by maintaining structural integrity at elevated temperatures through stable aluminosilicate network formation.

The optimized AASB formulations (MSWI-BA content: 0–12%, alkali > 3%, modulus > 1.25, NN/NK activators) exhibited significantly better compressive strength retention than OPC at both 400 °C and 800 °C, demonstrating superior thermal stability. This enhanced performance originates from fundamentally different degradation pathways: while OPC relies on partial recrystallization of CaO (derived from Ca(OH)_2_ decomposition) into larnite (Ca_2_SiO_4_) for limited strength retention at 800 °C [50], AASB systems maintain structural integrity through progressive dehydration and viscous sintering of their C-A-S-H/N-A-S-H gels, forming a continuous ceramic-like matrix above 600 °C [51]. Critically, AASB’s avoidance of Ca(OH)_2_ formation eliminates the expansive stresses and microcracking that severely compromise OPC’s high-temperature performance. Quantitative results demonstrate this advantage, with optimized AASB retaining 15–76% of initial strength at 800 °C compared to OPC’s typically inferior residual strength, highlighting AASB’s potential for structural applications requiring sustained mechanical performance under thermal exposure.

## 4. Discussion

### 4.1. Optimal Mix Proportion

**Figure 11:**
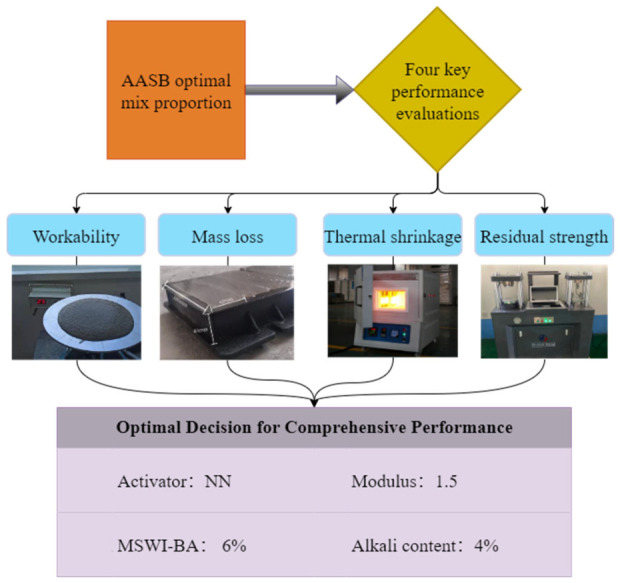
Evaluation process.

Based on the performance comparison analysis in Section 3, the optimal formulation with comprehensive performance was selected through the following evaluation process (Figure 11):

(i) Workability performance evaluation

Flowability and setting time are critical workability parameters for concrete engineering applications, as they directly affect construction placement under specific conditions. The results demonstrate that AASB achieves suitable flowability when containing less than 6% MSWI-BA content, more than 4% alkali content, a modulus greater than 1.50, and using NN or KN activators. For appropriate initial/final setting times, the optimal parameters are less than 6% MSWI-BA content, 2% or 4% alkali content, modulus of 0.75, and an NH activator.

(ii) Thermal mass loss analysis

Mass loss after high-temperature exposure serves as an important indicator for evaluating hydration product quantity and thermal resistance. When the MSWI-BA content ranges from 0% to 12%, alkali content from 2% to 6%, and modulus from 0.75 to 1.75, and using all activators except KH or KK groups, AASB shows comparable mass loss to OPC after 400 °C and 800 °C exposure, indicating excellent hydration product retention and thermal stability.

(iii) Thermal shrinkage characteristics

Thermal shrinkage after high-temperature treatment provides another key parameter for assessing hydration products and thermal resistance. With MSWI-BA content of 0–12%, alkali content of 2–6%, modulus of 0.75–1.75, and all activator types, AASB exhibits significantly higher thermal shrinkage than OPC at both 400 °C and 800 °C. However, it should be noted that this shrinkage will be substantially reduced when aggregates are incorporated in concrete mixtures.

(iv) Mechanical strength performance

Flexural and compressive strength represent fundamental requirements for concrete applications, while their normalized values after high-temperature exposure directly reflect thermal resistance. For normalized flexural strength superior to OPC and other AASB groups after 400 °C and 800 °C exposure, the optimal parameters are MSWI-BA content 0–12%, alkali content 2–4%, modulus 1.50, and all activators except the NH group. Regarding compressive strength, the best performance occurs with MSWI-BA content of 0–12%, alkali content of 3–6%, a modulus of 1.50, and all activators except NN or KK groups.

Consequently, the AASB prepared with sodium silicate solution modified by NaOH (NN-6), containing 4% alkali equivalent, 6% MSWI-BA content, and a 1.5 modulus, demonstrates optimal comprehensive performance. This optimal mixture is determined by a comprehensive evaluation of multiple performance indicators, including workability, thermal stability, and residual mechanical properties, rather than an independent analysis of single variables. It presents appropriate flowability and setting time, acceptable high-temperature mass loss and thermal shrinkage, as well as the highest normalized flexural and compressive strength after thermal exposure.

### 4.2. Economic Benefits

A detailed economic analysis of the optimized AASB system reveals a marginally higher unit cost (51.65 USD/ton) compared to conventional OPC (49.82 USD/ton), representing a 3.7% premium under current market conditions, as shown in Table 5, Table 6 and Table 7. This cost structure reflects distinct material pricing hierarchies, where sodium silicate activator (modulus 1.5) dominates AASB expenses at ~185.71 USD/ton, accounting for approximately 60% of total material costs. Other components follow traditional valuation patterns, with OPC priced at 67.14 USD/ton (market range: 54.29–80 USD/ton), BFS at intermediate levels, and subsidized MSWI-BA at 5 USD/ton through government circular economy incentives. The analysis highlights how activator chemistry currently drives AASB’s cost position, though the incorporation of waste-derived MSWI-BA demonstrates effective cost mitigation through industrial symbiosis strategies.

Beyond immediate cost metrics, AASB presents compelling economic and environmental value drivers that promise improved competitiveness at scale. First, according to industrial production experience, commercial production of activators is projected to potentially reduce costs by 20–30% through manufacturing optimization and bulk procurement. Second, the system avoids clinker production responsible for 8% of global CO_2_ emissions, yielding both carbon credit opportunities and protection against future carbon taxation [52]. Third, MSWI-BA integration exemplifies circular economy implementation, simultaneously reducing landfilling costs (typically 14.29–21.43 USD/ton) while creating value from waste streams [53]. These factors, combined with AASB’s demonstrated technical performance advantages in high-temperature applications, position it as a transformative alternative to OPC as sustainable construction policies mature. The current modest cost premium thus represents a transitional challenge rather than a fundamental limitation, with multiple pathways available to achieve cost parity while delivering superior lifecycle sustainability.

### 4.3. Environmental Benefits

A comprehensive life-cycle assessment reveals stark differences in carbon footprints between AASB and OPC systems, as shown in Table 8. While conventional OPC production emits 845 kg CO_2_/ton, primarily from limestone calcination (CaCO_3_ → CaO + CO_2_) and fossil fuel combustion AASB achieves a remarkable 66.95% reduction (208.10 vs. 629.68 kg CO_2_/ton paste). This dramatic decrease stems from three key factors: (i) utilization of carbon-neutral industrial byproducts (BFS and MSWI-BA) that avoid dedicated production emissions; (ii) minimized dosage (15–20%) of sodium silicate activator (1222 kg CO_2_/ton) through optimized formulation; and (iii) near-zero emission contribution from water (0.2 kg CO_2_/ton). The results align with global studies documenting 40–80% CO_2_ reductions for AAMs versus OPC [54]. Crucially, AASB’s environmental superiority derives from eliminating clinker production (responsible for 90% of OPC’s emissions) while valorizing challenging waste streams like MSWI-BA, a material with limited recycling pathways that would otherwise incur landfilling emissions and potential heavy metal leaching risks [34].

The study demonstrates AASB’s potential to revolutionize decarbonization in China’s construction sector (15–20% of national emissions) through three synergistic mechanisms. First, its technical compatibility with existing infrastructure enables seamless integration, avoiding the inertia of system-wide overhauls. Second, economic viability is enhanced through waste utilization subsidies and projected 20–30% cost reductions at commercial scale. Third, at full deployment, AASB could abate ~500 million tons of annual global cement emissions, directly supporting Intergovernmental Panel on Climate Change (IPCC) recommendations for low-carbon building materials. This positions AASB as a strategic solution aligned with multiple United Nations (UN) Sustainable Development Goals: SDG 9 (sustainable industry) through waste valorization and SDG 13 (climate action) via emission mitigation. As China progresses toward its 2030/2060 carbon targets, AASB represents a ready-to-implement technology that bridges environmental and industrial policy objectives while addressing the critical waste-to-resource challenge in urban ecosystems.

### 4.4. Comprehensive Comparison with OPC

**Figure 12:**
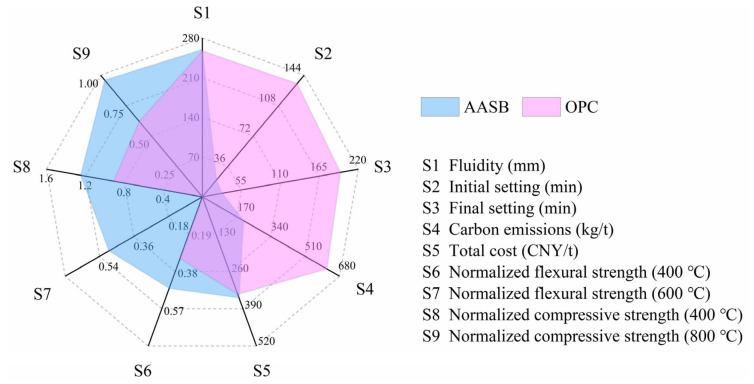
Comprehensive performance comparison diagram.

A systematic evaluation of AASB versus OPC reveals distinct advantages across multiple metrics, as shown in Figure 12. While both systems demonstrate comparable workability (AASB: 260 mm vs. OPC: 257.5 mm flow spread), AASB exhibits significantly faster setting behavior (initial/final: 20/28 min) compared to OPC (135/195 min) due to accelerated geopolymerization kinetics. This rapid setting, though potentially moderated by aggregate effects in concrete applications, offers distinct advantages for precast construction. Thermal performance testing demonstrates AASB’s superior resilience, maintaining 93.55% greater normalized flexural strength at 400 °C (0.6 vs. OPC’s 0.31) and retaining structural integrity at 600 °C (0.48) where OPC fails completely. Similarly, AASB’s compressive strength retention at 400 °C (1.25) and 800 °C (0.96) surpasses OPC by 37.36% and 54.84%, respectively, attributable to its stable C-A-S-H/N-A-S-H gel networks that resist thermal degradation, in contrast to OPC’s Ca(OH)_2_ decomposition-induced microcracking.

The environmental assessment highlights AASB’s dramatic 66.95% carbon footprint reduction (208.10 kg CO_2_/t) compared to OPC (629.68 kg CO_2_/t), achieved through the elimination of clinker production and the valorization of industrial byproducts (BFS and MSWI-BA). This performance aligns with IPCC’s low-carbon material recommendations. Economically, AASB’s current cost premium (51.65 vs. 49.82 USD/t for OPC) reflects sodium silicate expenses, yet is offset by three strategic benefits: (i) waste management savings (MSWI-BA subsidy: 5 USD/t) with associated environmental risk mitigation; (ii) compliance with China’s carbon neutrality targets; and (iii) enhanced fire resistance for critical infrastructure. Projected scale effects in activator production and policy support for circular economy practices are anticipated to bridge the current cost gap. This comprehensive profile positions AASB as a technically superior and environmentally transformative alternative to OPC, directly advancing UN Sustainable Development Goals for industrial innovation (SDG 9) and climate action (SDG 13).

## 5. Conclusions

This study systematically investigates the performance of alkali-activated slag binder (AASB) incorporating municipal solid waste incineration bottom ash (MSWI-BA), focusing on the effects of MSWI-BA content, alkali content, WG modulus, and activator type. Through comprehensive experimental analysis, the optimized formulation demonstrates superior high-temperature performance compared to ordinary Portland cement (OPC), while maintaining excellent workability and sustainability. The principal findings are summarized as follows:

(1) The NN-6 formulation (6% MSWI-BA, 4% Na_2_O, Ms = 1.5) achieves an optimal balance between workability (260 mm flow diameter) and thermal stability, retaining 93.55% higher flexural strength at 400 °C and 76% compressive strength at 800 °C compared to OPC, attributed to its stable C-A-S-H/N-A-S-H gel networks and refined pore structure.
(2) The incorporation of MSWI-BA reduces the cost of raw precursor materials by 88% (5 vs. 42.86 USD/ton) and realizes effective heavy metal immobilization via chemical encapsulation in the aluminosilicate matrix. These results demonstrate obvious economic and environmental advantages while maintaining comparable technical performance to conventional binders.
(3) Life cycle assessment quantifies that AASB achieves a 66.95% lower carbon footprint (208.10 vs. 629.68 kg CO_2_/ton) than OPC, mainly owing to the avoidance of clinker calcination and the valorization of solid wastes. This confirms that the prepared AASB is a low-carbon and sustainable alternative binder for green construction applications.

Limitations: This study focuses on the performance of AASB paste; the effects of coarse and fine aggregates on workability, high-temperature resistance, and long-term durability require further verification.

Future research: Further studies will be conducted on the long-term durability, practical engineering applications, and large-scale production economic benefits of MSWI-BA modified AASB to promote its engineering application.

## Figures and Tables

**Table 1:** Chemical compositions of precursor (%) [12].

Precursor	SiO_2_	Fe_2_O_3_	MgO	Al_2_O_3_	CaO	K_2_O	Others
BFS	31.59	1.13	5.85	13.99	39.91	0.66	6.87
MSWI-BA	21.68	6.29	2.10	5.76	54.21	1.77	8.19

**Table 2:** Water absorption and specific surface area of precursor [12].

Precursor	Water Absorption (%)	Specific Surface Area (m^2^/kg)
BFS	40.4	429
MSWI-BA	84.1	11,467

**Table 3:** Mix proportions of AASB (g).

Variable	Group	WG	BFS	MSWI-BA	Water
MSWI-BA content	NN-0	984.39	3900	0	896.90
	NN-3		3783	117	
	NN-6		3666	234	
	NN-9		3549	351	
	NN-12		3432	468	
Alkali content	NN2	492.21	3432	468	1130.94
	NN3	738.33			1013.90
	NN4	984.39			896.90
	NN5	1230.54			779.84
	NN6	1476.66			662.81
WG moduli	Ms0.75	592.89	3432	468	1135.57
	Ms1.00	723.30			1056.11
	Ms1.25	853.88			976.48
	Ms1.50	984.39			896.90
	Ms1.75	1114.86			817.40

Note: WG stands for water glass, referring to sodium silicate or potassium silicate activators; BFS represents granulated blast furnace slag.

**Table 4:** Mix proportion of AASB with different activator types (g).

Group	NH	KH	NS/NH	KS/KH	NS/KH	KS/NH	BFS	MSWI-BA	Water
NH	201.3								1435.5
KH		185.9							1430.1
NN			985.0				3432	468	896.9
KK				652.1					1125.2
NK					802.5				1011.6
KN						765.2			1058.0

Note: NH represents NaOH; KH represents KOH; NN denotes sodium silicate with modulus 1.5 adjusted by NaOH; KK denotes potassium silicate with modulus 1.5 adjusted by KOH; NK denotes sodium silicate with modulus 1.5 adjusted by KOH; KN denotes potassium silicate with modulus 1.5 adjusted by NaOH. NS represents sodium silicate activator; KS stands for potassium silicate activator. All WG systems maintain a constant modulus of 1.5.

**Table 5:** Mix proportion of AASB and OPC (g).

Group	WG	BFS	MSWI-BA	Water	Cement
AASB	170.27	634.11	40.48	155.14	/
OPC	/	/	/	259.26	740.74

**Table 6:** Cost list of OPC per ton.

Ingredient	Dosage (kg)	Unit Price (USD/kg)	Price (USD)	Total Cost (USD/t)
Cement	740.74	0.0671	49.74	49.82
Water	259.26	0.00034	0.09	

**Table 7:** Cost list of AASB per ton.

Ingredient	Dosage (kg)	Unit Price (USD/kg)	Price (USD)	Total Cost (USD/t)
BFS	634.11	0.0304	19.30	51.65
MSWI-BA	40.48	0.005	0.203	
WG	170.27	0.186	31.621	
Water	155.14	0.0034	0.531	

**Table 8:** Carbon emission coefficient of raw materials.

Ingredient	OPC Paste (1 t)			AASB Paste (1 t)		
	Dosage (kg)	Carbon Emission Coefficient (kg CO_2_/kg)	Carbon Emissions (kg)	Dosage (kg)	Carbon Emission Coefficient (kg CO_2_/kg)	Carbon Emissions (kg)
Cement	740.74	0.85	629.63	/	/	/
Water	259.26	0.0002	0.05	155.14	0.0002	0.03
BFS	/	/	/	634.11	0	0
MSWI-BA	/	/	/	40.48	0	0
WG	/	/	/	170.27	1.222	208.07
In total	/	/	629.68	/	/	208.10

## Data Availability

The original contributions presented in this study are included in the article. Further inquiries can be directed to the corresponding authors.

## Abbreviations

The following abbreviations are used in this manuscript:

OPC	Ordinary Portland cement
MSWI-BA	municipal solid waste incineration bottom ash
MSWI	municipal solid waste incineration
AAMs	Alkali-activated materials
BFS	Blast furnace slag
C-A-S-H	Calcium-aluminosilicate-hydrate
XRD	X-ray diffraction
WG	Water glass
AASB	MSWI-BA-incorporated alkali-activated slag paste
XRF	X-ray fluorescence
